# Distributed Virtual Inertia Control Strategy for Multi-Virtual Synchronous Machine Parallel System Based on Neighbor Communication

**DOI:** 10.3390/s25092855

**Published:** 2025-04-30

**Authors:** Ge Cao, Hanbing Wu, Yao Liu, Qiang Ren

**Affiliations:** 1 School of Electrical Engineering, Xi’an University of Technology, Xi’an 710054, China; gcao@xaut.edu.cn; 2 Electric Power Research Institute, State Grid Shaanxi Electric Power Co., Ltd., Xi’an 710100, China; liuyaoseu@163.com (Y.L.); 15376800333@163.com (Q.R.)

**Keywords:** virtual synchronous machine, frequency oscillation, small signal model, virtual inertia control, energy function method

## Abstract

As a typical grid-forming control method, virtual synchronous generator (VSG) control enhances system inertia but introduces frequency oscillation issues. This problem becomes particularly severe in multi-VSG parallel systems when inconsistent virtual inertia exists among power sources, significantly compromising the security and stability of power system operation. Virtual inertia control can directly regulate the rate of change of frequency (RoCoF) under constant torque difference conditions to suppress frequency oscillations, offering faster response characteristics. Therefore, this paper proposes a distributed virtual inertia control strategy for multi-VSG parallel systems. First, a small-signal model of the multi-machine parallel system is established, and its small-signal stability is demonstrated. Second, a neighbor-communication-based distributed virtual inertia coordination control method is proposed. Through neighbor information exchange and local decision-making, this method enables dynamic adjustment of each unit’s virtual inertia, driving frequency synchronization among all units in the system. This effectively suppresses post-disturbance frequency oscillations and enhances the dynamic performance of low-inertia power systems. Furthermore, the stability of the proposed control strategy is rigorously proven through the construction of a Lyapunov energy function. Finally, MATLAB/Simulink simulations verify that the proposed virtual inertia control strategy can effectively mitigate frequency oscillations while reducing their settling time.

## 1. Introduction

The integration of distributed generation (DG) systems, primarily wind and solar power, into the grid has significantly reduced the inertia of power systems [1]. This reduction makes the system more susceptible to frequency oscillations when subjected to disturbances, presenting new challenges to the safe and stable operation of power systems [2].

In conventional power systems, synchronous machines provide inertia support. In modern power systems, grid-forming converter-based DG autonomously establishes voltage and frequency to provide grid support capabilities [3]. Among these technologies, the virtual synchronous generator (VSG) control [4] introduces rotor motion equations into the converter to emulate the external characteristics of synchronous generators, thereby endowing DG with corresponding inertia and damping [5]. While this enhances power system inertia, it simultaneously introduces active power–frequency oscillations [6]. Compared to single-machine systems, parallel-operated multi-VSG systems exhibit more severe active power–frequency oscillations during disturbances [7].

The stability analysis of multi-VSG systems is typically conducted using state–space methods [8]. The state–space approach employs root locus techniques for systematic stability analysis of the control parameters. Reference [9] simplified the model to develop a novel third-order control model that demonstrates high accuracy in power system stability analysis. A comprehensive state–space model was established in [10], while [11] developed a small-signal model for parallel multi-VSG systems to investigate the mechanism of active power output oscillations. Furthermore, [12] proposed a generalized small-signal model for multi-machine VSG systems, enabling precise analysis of how control parameters influence frequency stability. Research indicates that virtual inertia [13], damping coefficients, active power droop coefficients, outer-loop control parameters, and line impedance characteristics [14] collectively impact the stability of multi-machine systems [15].

Current research on frequency oscillation suppression in multi-VSG parallel systems has primarily focused on two control strategy categories: power control strategies and dynamic damping/inertia adjustment approaches. Power control strategies achieve oscillation suppression through active power output regulation, though their effectiveness is limited by the inherent time delay between control signal initiation and power output adjustment, particularly during rapid load transitions where they may fail to meet oscillation suppression requirements. A centralized control method based on the center of inertia concept was proposed in [16] to adjust VSG input power for suppressing power fluctuations and mitigating frequency oscillations. A distributed communication framework is developed in [17] to establish angular frequency references through load factor interaction among multiple VSGs, where active power compensation is generated from frequency deviations for frequency restoration purposes. Through system impedance modeling, it is demonstrated in [18] that the fundamental oscillation mechanism is constituted by the interplay between system inertia and damping characteristics, which leads to the classification of existing suppression methods into damping control and inertia control categories. A decentralized mutual damping approach is introduced in [19], where damping terms are enhanced through power derivatives, while frequency differences among units are incorporated via consensus control to accelerate transient frequency convergence. A dynamic virtual damping control technique is presented in [20], where damped low-frequency oscillation components are superimposed onto electromagnetic torque to enhance system damping during transient conditions for active power–frequency oscillation suppression. A novel control strategy combining consensus-based mutual damping with model predictive control is proposed in [21] to simultaneously achieve oscillation suppression and optimal economic active power dispatch.

The damping control method indirectly regulates the rate of frequency change through torque difference compensation, thereby suppressing the magnitude of frequency oscillations. In contrast to damping control, virtual inertia control directly modulates the frequency change rate through dynamic virtual inertia adjustment without requiring torque compensation. This approach enables faster response to smooth frequency fluctuations while maintaining a relatively simpler control architecture that is more renewable-energy-friendly. Regarding dynamic inertia adjustment, a fuzzy control-based technique is developed in [22], where governor output power is adaptively regulated through fuzzy logic to reinforce system inertia for effective load fluctuation mitigation. An adaptive virtual inertia control scheme is developed in [23], which employs decentralized local control to dynamically adjust rotational inertia during load variations, achieving effective frequency oscillation suppression while inherently lacking global system coordination capability due to its decentralized architecture.

The remainder of this paper is structured as follows: Section 2 presents the small-signal modeling method of the multi-VSG parallel system, establishing the theoretical foundation for the proposed control strategy. Section 3 introduces the distributed virtual inertia control strategy based on neighbor communication, describing its fundamental principles and implementation details. Section 4 provides a rigorous stability analysis for the proposed strategy, utilizing the Lyapunov energy function method. Section 5 demonstrates the effectiveness and advantages of the proposed strategy through comprehensive simulation studies in MATLAB/Simulink, including performance comparisons under various scenarios. Finally, Section 6 summarizes the main findings and conclusions of this research.

## 2. Small-Signal Modeling of Multi-Virtual Synchronous Machine (VSG) Parallel System

### 2.1. Virtual Synchronous Generator (VSG) Parallel System

**Figure 1:**
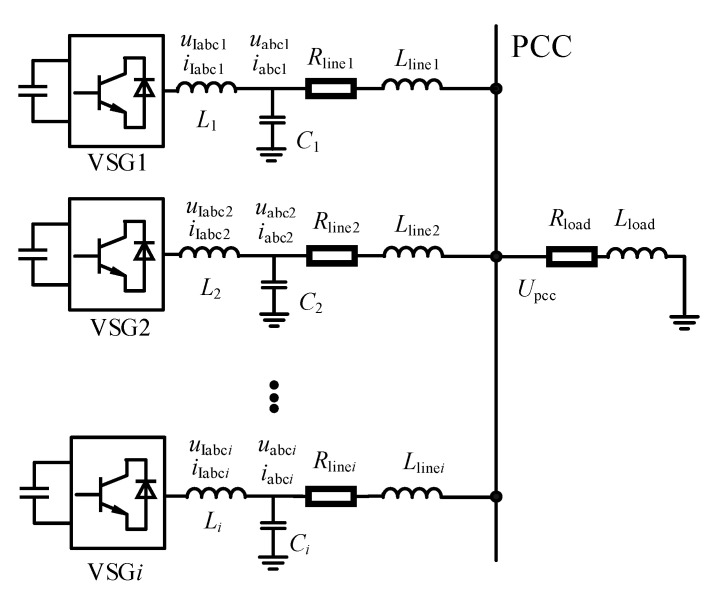
Multi-VSG parallel system.

The multi-machine parallel system based on VSG technology adopts a common bus parallel architecture, as illustrated in Figure 1. In this system, *N* VSG units (*i* = 1, 2, …, *N*) operate in parallel on an AC bus through distributed connection lines. The output terminal of each VSG unit is connected to the point of common coupling via an equivalent impedance. The output currents *I_i_* of all VSG units are vectorially summed at the PCC, forming the total system current as *I*_total_ = ∑*I_i_*.

In a multi-VSG parallel system, *u*_Iabc*i*_ and *i*_Iabc_*_i_* represent the port output voltage and output current of the *ith* VSG unit, respectively, while *u*_abc*i*_ and *i*_abc*i*_ denote the filtered output voltage and current after passing through the LC filter. The parameters *L_i_* and *C_i_* correspond to the filter inductance and capacitance of the *ith* VSG unit, which are designed to suppress high-frequency harmonic components and improve power quality. *R*_line,*i*_ and *L*_line,*i*_ represent the equivalent resistance and inductance of the transformer and transmission line between the output terminal of the *ith* VSG unit and the point of common coupling (PCC), which significantly impact power flow distribution and system stability. Additionally, *R*_load_ and *L*_load_ denote the equivalent resistance and inductance of the load, determining the overall impedance characteristics of the system. The PCC voltage *U*_pcc_ serves as a critical indicator for evaluating the system’s voltage regulation capability and stability.

**Figure 2:**
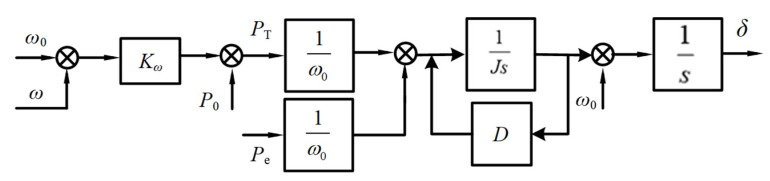
Single-VSG control structure.

A typical control structure of a VSG is illustrated in Figure 2. The control strategy primarily consists of power calculation, active power–frequency control, voltage–current dual-loop control, and pulse width modulation technology. The power outer-loop control of the VSG comprises two critical components: the active power–frequency control for system frequency regulation and dynamic response enhancement and the reactive power–voltage control responsible for maintaining bus voltage stability and optimizing reactive power distribution. The VSG’s active power–frequency regulation is achieved by incorporating the rotor motion equation of synchronous generators, along with the *P*-*ω* droop control strategy. The rotor motion equation describes the inertia and damping characteristics of synchronous machines, with its mathematical formulation given in Equation (1). Simultaneously, the system’s mechanical power regulation is provided by the *ω*-*P* droop control strategy, and its mathematical expression is presented in Equation (2):

(1) $$\left\{\begin{array}{l} J\frac{d\omega }{dt}=\frac{P_{T}}{\omega_{0}}-\frac{P_{e}}{\omega_{0}}-D\Delta \omega \\ \frac{d\delta }{dt}=\omega \end{array}\right.$$

(2) $$P_{T}=P_{0}+K_{\omega }(\omega_{0}-\omega )$$

In this context, *P*_0_ and *ω*_0_ represent the reference power and reference angular frequency of the VSG under rated operating conditions, while *P*_T_ and *ω* denote the mechanical power and actual output angular frequency during the VSG’s real-time operation. *K_ω_* is the active power–frequency droop coefficient of the VSG. The parameter *J* represents the virtual rotational inertia of the VSG, which reflects the system’s capability to respond to frequency disturbances, while *D* is the virtual damping coefficient, responsible for regulating the dynamic characteristics and stability of the system. *P*_e_ denotes the electromagnetic power output of the VSG, which is obtained by measuring the three-phase output voltage and current and performing active power calculations. Additionally, *δ* represents the phase angle of the VSG, Δ*ω* is the angular frequency deviation, and *t* denotes the system’s operational time.

The reactive power–voltage control of the VSG simulates the reactive voltage regulation characteristics of an exciter by introducing *Q*-*U* droop control. This reactive power–voltage control strategy can be specifically expressed by Equation (3):

(3) $$U=U_{0}+(Q_{0}-Q)K_{Q}$$

where *U*_0_ and *Q*_0_ represent the terminal voltage and reactive power output of the VSG under rated operating conditions, respectively. *U* and *Q* denote the terminal voltage and reactive power output during actual operation, while *K_Q_* is the reactive power droop coefficient of the VSG.

### 2.2. VSG Parallel System Small-Signal Modeling

**Figure 3:**
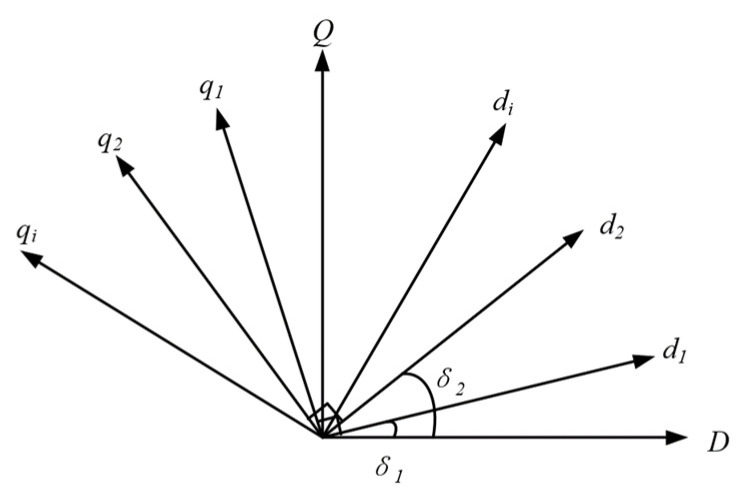
Reference coordinate transformation.

Based on the topology of the multi-VSG parallel system, small-signal modeling was conducted. Considering that the dq reference coordinate systems of different VSGs are not identical, one VSG unit’s coordinate system was selected as the reference coordinate system DQ. The coordinate systems of the other VSG units were then transformed into this reference frame. The coordinate transformation from the dq axis to the DQ axis is illustrated in Figure 3. The transformation process of the rotating dq coordinate system of the *ith* VSG unit to the reference DQ coordinate system is expressed as:

(4) $$\left[\begin{array}{l} x_{D}\\ x_{Q} \end{array}\right]=\left[\begin{array}{cc} \cos\delta_{i} & -\sin\delta_{i}\\ \sin\delta_{i} & \cos\delta_{i} \end{array}\right]\left[\begin{array}{l} x_{di}\\ x_{qi} \end{array}\right]$$

The small-signal model corresponding to Equation (4) is expressed as:

(5) $$\begin{array}{l} \left[\begin{array}{c} \Delta x_{D}\\ \Delta x_{Q} \end{array}\right]=\left[\begin{array}{cc} \cos\delta_{i} & -\sin\delta_{i}\\ \sin\delta_{i} & \cos\delta_{i} \end{array}\right]\left[\begin{array}{c} \Delta x_{di}\\ \Delta x_{qi} \end{array}\right]\\ +\left[\begin{array}{c} -X_{di}\sin\delta_{i}-X_{qi}\cos\delta_{i}\\ X_{di}\cos\delta_{i}-X_{qi}\sin\delta_{i} \end{array}\right]\Delta \delta_{i} \end{array}$$

The inverse transformation process from the reference DQ coordinate system to the rotating dq coordinate system of the *ith* VSG unit is expressed as:

(6) $$\left[\begin{array}{l} X_{di}\\ X_{qi} \end{array}\right]=\left[\begin{array}{cc} \cos\delta_{i} & \sin\delta_{i}\\ -\sin\delta_{i} & \cos\delta_{i} \end{array}\right]\left[\begin{array}{l} X_{D}\\ X_{Q} \end{array}\right]$$

The small-signal model corresponding to Equation (6) is expressed as:

(7) $$\begin{array}{l} \left[\begin{array}{c} \Delta x_{di}\\ \Delta x_{qi} \end{array}\right]=\left[\begin{array}{cc} \cos\delta_{i} & \sin\delta_{i}\\ -\sin\delta_{i} & \cos\delta_{i} \end{array}\right]\left[\begin{array}{c} \Delta x_{D}\\ \Delta x_{Q} \end{array}\right]\\ +\left[\begin{array}{c} -X_{D}\sin\delta_{i}+X_{Q}\cos\delta_{i}\\ -X_{D}\cos\delta_{i}-X_{Q}\sin\delta_{i} \end{array}\right]\Delta \delta_{i} \end{array}$$

Taking the rotating coordinate system of the first VSG unit as the reference coordinate system, the phase angle difference between the other VSG units and the first VSG unit is expressed as:

(8) $$\delta_{i}=\int \left(\omega_{i}-\omega_{1}\right)dt$$

The small-signal model after processing Equation (8) is expressed as:

(9) $$\Delta \delta_{i}=\Delta \omega_{i}-\Delta \omega_{1}$$

The power control loop of the VSG consists of a power calculation, as well as active power–frequency control and reactive power–voltage control in VSG regulation. The power calculation process is expressed by Equation (10), and its linearized form is given by Equation (11). The implementation of the VSG control strategy is represented by Equations (1)–(3), while its linearized form is expressed by Equation (12):

(10) $$\left\{\begin{array}{c} P=\frac{3}{2}(u_{d}i_{d}+u_{q}i_{q})\\ Q=\frac{3}{2}(u_{q}i_{d}-u_{d}i_{q}) \end{array}\right.$$

In the equation, *P* and *Q* represent the average active power and reactive power, respectively. *u*_d_ and *u*_q_ denote the output voltages of the inverter transformed to the d-axis and q-axis through the park transformation. *i*_d_ and *i*_q_ are the output currents of the inverter in the d-axis and q-axis, respectively:

(11) $$\left\{\begin{array}{l} \Delta P=\frac{3}{2}\left(\Delta u_{d}I_{d}+U_{d}\Delta i_{d}+\Delta u_{q}I_{q}+U_{q}\Delta i_{q}\right)\\ \Delta Q=\frac{3}{2}\left(\Delta u_{q}I_{d}+U_{q}\Delta i_{d}-\Delta u_{d}I_{q}-U_{d}\Delta i_{q}\right) \end{array}\right.$$

(12) $$\begin{array}{l} \Delta \dot{\omega }=-\frac{D\omega_{0}+K_{\omega }}{J\omega_{0}}\Delta \omega -\frac{1}{J\omega_{0}}\Delta P\\ \left\{\begin{array}{l} \Delta E_{d}=-K_{Q}\Delta Q\\ \Delta E_{q}=0 \end{array}\right. \end{array}$$

Considering that the dual-loop voltage and current control has fast response and high accuracy, small-signal modeling of the voltage and current loops was not performed. It was assumed that the reference voltage output from the power control loop is approximately equal to the modulation voltage output from the voltage and current control loops, which is also approximately equal to the output voltage of the VSG unit.

The rationale behind this simplification lies in the fact that the inner control loops are typically designed with high-bandwidth controllers (often in the kilohertz range), which enables them to respond almost instantaneously. Compared to the outer-loop dynamics of power and frequency control, which typically operate at frequencies of a few hertz, the inner loops can be regarded as being in a quasi-steady-state.

Moreover, the main focus of this study is on the analysis and suppression of frequency oscillations and active power dynamics, which predominantly occur in the low- to mid-frequency range. Within this frequency range, the influence of the voltage and current inner control loops on system-level stability is relatively limited. Therefore, to simplify the modeling process and concentrate on the dominant dynamics related to the control objectives, the inner loops were omitted from the small-signal model.

Nevertheless, to ensure that the essential electromagnetic dynamics between the converter and the external grid/load were preserved, the differential equations of the LC output filter were explicitly incorporated into the state–space model (see Equation (13)). This allowed for an accurate representation of the dynamic behavior at the converter output interface:

(13) $$\left\{\begin{array}{l} L\frac{di_{\mathrm{Id}}}{dt}=L\omega i_{\mathrm{Iq}}+u_{\mathrm{Id}}-u_{d}\\ L\frac{di_{\mathrm{Iq}}}{dt}=-L\omega i_{\mathrm{Id}}+u_{\mathrm{Id}}-u_{q}\\ C\frac{du_{d}}{dt}=C\omega u_{q}+i_{\mathrm{Id}}-i_{d}\\ C\frac{du_{q}}{dt}=-C\omega u_{d}+i_{\mathrm{Iq}}-i_{q} \end{array}\right.$$

The mathematical model of the line impedance is expressed as:

(14) $$\left\{\begin{array}{l} L_{\mathrm{line}}\frac{di_{d}}{dt}=-R_{\mathrm{line}}i_{d}+L_{\mathrm{line}}\omega i_{q}+u_{d}-u_{\mathrm{pccd}}\\ L_{\mathrm{line}}\frac{di_{q}}{dt}=-R_{\mathrm{line}}i_{q}-L_{\mathrm{line}}\omega i_{d}+u_{q}-u_{\mathrm{pccq}} \end{array}\right.$$

Considering an RL load as a typical load in a microgrid, the mathematical model representing the connection from the PCC to the load is expressed as:

(15) $$\left\{\begin{array}{c} L_{\mathrm{load}}\frac{di_{\mathrm{loadD}}}{dt}=-R_{\mathrm{load}}i_{\mathrm{gD}}+L_{\mathrm{load}}\omega_{l}i_{\mathrm{loadQ}}+u_{\mathrm{pccD}}\\ L_{\mathrm{load}}\frac{di_{\mathrm{loadQ}}}{dt}=-R_{\mathrm{load}}i_{\mathrm{gQ}}-L_{\mathrm{load}}\omega_{l}i_{\mathrm{loadD}}+u_{\mathrm{pccQ}} \end{array}\right.$$

In the equation, *i*_loadDQ_ represents the current flowing through the RL load in the DQ reference coordinate system.

By combining Equations (9) and (11)–(15), the state variable matrix for a single VSG is defined as Δ*x_i_*, which is expressed as:

(16) $$\Delta {\dot{x}}_{\mathrm{sys}}=A_{\mathrm{sys}}\Delta x_{\mathrm{sys}}$$

where the state variable matrix for multiple VSGs, Δ*x*_sys_, is expressed as:

(17) $$\Delta x_{\mathrm{sys}}=[\Delta x_{1}\;\Delta x_{2}\;\Delta x_{3}\cdots \Delta x_{i}\;\Delta i_{\mathrm{loadD}}\;\Delta i_{\mathrm{loadQ}}]$$

For a VSG parallel system with *i* = 5, all eigenvalues of the five-VSG parallel system under this operating condition are located in the left half-plane of the coordinate system, indicating that the system is stable under small disturbances.

The small-signal model developed in this section provides a detailed state–space representation of the multi-VSG parallel system, incorporating the control loops, power circuit components, and inter-unit coupling. This model serves as a theoretical foundation for analyzing the system’s dynamic behavior and forms the structural basis for the subsequent control design. In the next section, a distributed virtual inertia control strategy is proposed, building upon the modeled dynamics to enhance frequency coordination and suppress oscillations in a fully decentralized manner.

## 3. Distributed Virtual Inertia Control Strategy

**Figure 4:**
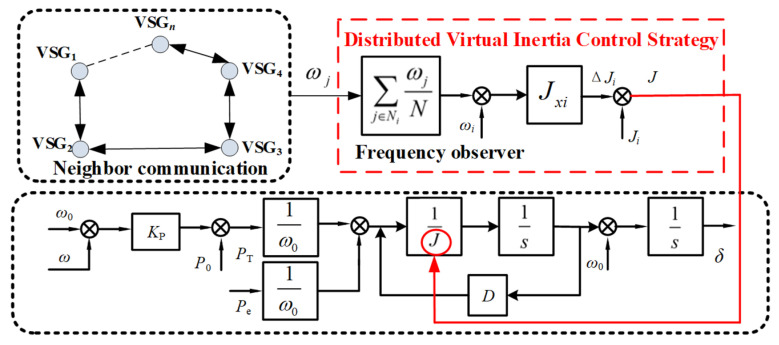
Virtual inertia control strategy.

This paper proposes a distributed virtual inertia control strategy based on neighbor communication. By leveraging neighbor communication, quasi-global sharing of frequency information is achieved, allowing the introduction of an additional inertia term to adjust the virtual inertia of the local VSG. This helps suppress frequency oscillations after disturbances. The control block diagram is shown in Figure 4.

By introducing an additional inertia term, the improved rotor motion equation is expressed as Equation (18):

(18) $$\left\{\begin{array}{l} (J_{i}+\Delta J_{i})\frac{d\omega_{i}}{dt}=\frac{P_{Ti}}{\omega_{i}}-\frac{P_{ei}}{\omega_{i}}-D_{i}\Delta \omega_{i}\\ \Delta J_{i}=J_{xi}(\omega_{i}-{\hat{\omega }}_{i})\mathrm{sign}(\frac{d\omega_{i}}{dt}) \end{array}\right.$$

In the equation, *J_i_* represents the virtual inertia of the *ith* VSG (virtual synchronous generator) unit in the system, and Δ*J_i_* denotes the introduced additional inertia term. *J_xi_* is defined as the additional inertia coefficient of the *ith* VSG, *ω_i_* is the angular frequency of the *ith* VSG, and ${\hat{\omega }}_{i}$ is the reference value of the angular frequency of the *ith* VSG obtained through neighbor information interaction. The function sign is the sign function, which takes the value 1 when d*ω_i_/*d*t* > 0, 0 when d*ω_i_/*d*t* = 0, and −1 when d*ω_i_/*d*t* < 0. By adjusting the additional inertia term Δ*J_i_*, the virtual inertia of the *ith* VSG can be modified, thereby altering d*ω_i_/*d*t* to achieve frequency oscillation suppression.

In a multi-VSG parallel system, if each VSG aims to obtain an accurate reference value of angular frequency for local computation of the additional inertia term, it is necessary to acquire the angular frequency variations of neighboring VSGs in real time. To achieve this goal, sensors were typically deployed in the system to observe the angular frequency of each VSG. These sensors can measure the angular frequency *ω_i_* of each VSG in real time and transmit the data to the distributed control network.

To improve the dynamic performance during the frequency variation process, a distributed frequency state observer based on neighbor communication was designed within the additional inertia control strategy, as shown in Equation (19). This observer utilizes the angular frequency data collected by the sensors, combined with communication between neighboring VSGs, to dynamically estimate the reference angular frequency ${\hat{\omega }}_{i}$ for each VSG:

(19) $${\hat{\omega }}_{i}=\sum_{j\in N_{i}}\frac{\omega_{j}}{N}$$

In the equation, *ω_j_* represents the output angular frequency of the *jth* VSG adjacent to the *ith* VSG, *N_i_* denotes the set of neighbors defined for the *ith* VSG, and *N* represents the number of neighbors of the *ith* VSG.

Considering the neighbor communication architecture, which does not require a central control node and only relies on the exchange of angular frequency information obtained from sensors among VSGs, the angular frequency of each unit in the system can converge to a consistent value. This architecture has low requirements for communication and computational resources, requiring only minimal communication costs to observe and process the angular frequency information of neighboring VSGs. To achieve the observation of angular frequencies for multiple VSG units adjacent to the local VSG, this study ensured that the communication topology in the neighbor communication framework aligned with the physical topology of the actual multi-VSG system, where the angular frequency information is directly sourced from sensors. By integrating real-time data collected by sensors with neighbor communication, the system can achieve efficient and distributed frequency synchronization and control.

When a new load is connected to a multi-VSG parallel system during steady-state operation, the changes in the local angular frequency *ω_i_*, the average neighbor angular frequency ${\hat{\omega }}_{i}$, the rate of change of angular frequency d*ω_i_/*d*t*, and the virtual inertia *J_i_* of the *ith* unit over an oscillation cycle can be divided into the following four stages:

**Stage I:** The local angular frequency *ω_i_* and the average neighbor angular frequency ${\hat{\omega }}_{i}$ satisfy *ω_i_* < ${\hat{\omega }}_{i}$ and d*ω_i_/*d*t* < 0. To ensure that the output angular frequencies of all VSG units converge to a consistent value, the virtual inertia *J_i_* is increased to constrain the rate of change of angular frequency d*ω_i_/*d*t*. As a result, the local angular frequency *ω_i_* increases compared to the case without control, the absolute deviation |Δ*ω_i_*| decreases compared to the case without control, and *ω_i_* moves closer to the average neighbor angular frequency ${\hat{\omega }}_{i}$.

**Stage II:** The local angular frequency *ω_i_* and the average neighbor angular frequency ${\hat{\omega }}_{i}$ satisfy *ω_i_* < ${\hat{\omega }}_{i}$ and d*ω_i_/*d*t* > 0. To ensure that the output angular frequencies of all VSG units converge to a consistent value, the virtual inertia *J_i_* is decreased to increase the rate of change of angular frequency d*ω_i_/*d*t*. As a result, the local angular frequency *ω_i_* increases compared to the case without control, the absolute deviation |Δ*ω_i_*| decreases compared to the case without control, and *ω_i_* moves closer to the average neighbor angular frequency ${\hat{\omega }}_{i}$.

**Stage III:** The local angular frequency *ω_i_* and the average neighbor angular frequency ${\hat{\omega }}_{i}$ satisfy *ω_i_* > ${\hat{\omega }}_{i}$ and d*ω_i_/*d*t* > 0. To ensure that the output angular frequencies of all VSG units converge to a consistent value, the virtual inertia *J_i_* is increased to reduce the rate of change of angular frequency d*ω_i_/*d*t*. As a result, the local angular frequency *ω_i_* decreases compared to the case without control, moving closer to the average neighbor angular frequency ${\hat{\omega }}_{i}$.

**Stage IV:** The local angular frequency *ω_i_* and the average neighbor angular frequency ${\hat{\omega }}_{i}$ satisfy *ω_i_* > ${\hat{\omega }}_{i}$ and d*ω_i_/*d*t* < 0. To ensure that the output angular frequencies of all VSG units converge to a consistent value, the virtual inertia *J_i_* is decreased to increase the rate of change of angular frequency d*ω_i_/*d*t*. As a result, the local angular frequency *ω_i_* decreases compared to the case without control, moving closer to the average neighbor angular frequency ${\hat{\omega }}_{i}$.

After the system is subjected to a disturbance, a repeated oscillatory decay process occurs. Each oscillation cycle repeats the aforementioned four stages. During the disturbance process of the system, the proposed control strategy can take effect in all oscillatory decay processes, thereby effectively suppressing frequency oscillations.

## 4. Stability Analysis of the System Using the Energy Function Method

For an n-VSG parallel system, where each VSG is equivalent to a PV node, the active power output of the *ith* VSG is given by:

(20) $$P_{ei}=U_{i}^{2}G_{ii}+U_{i}\overset{j=n}{\underset{\begin{array}{l} j=1\\ j\neq i \end{array}}{\sum }}U_{j}(\left|G_{ij}\right|\cos\delta_{ij}+\left|B_{ij}\right|\sin\delta_{ij})$$

Assuming *δ_i_* − *δ_j_* ≈ 0,and cos*δ_ij_* ≈ 1, and defining the initial value of the output power as *P*_e*i*0_, Equation (20) can be expressed as Equation (21):

(21) $$\left\{\begin{array}{l} P_{\mathrm{ei}}=P_{ei0}+U_{i}\overset{j=n}{\underset{\begin{array}{l} j=1\\ j\neq i \end{array}}{\sum }}U_{j}B_{ij}\sin\delta_{ij}\\ P_{ei0}=U_{i}^{2}G_{ii}+U_{i}\overset{j=n}{\underset{\begin{array}{l} j=1\\ j\neq i \end{array}}{\sum }}U_{j}G_{ij} \end{array}\right.$$

Substituting Equation (21) into Equation (18) yields:

(22) $$\begin{array}{cc} (J_{i}+J_{xi}(\omega_{i}-{\hat{\omega }}_{i})\mathrm{sign}\frac{d\omega_{i}}{dt})\omega_{0}\frac{d\omega_{i}}{dt}=P_{Ti}-P_{ei0} & -\\ U_{i}\overset{j=n}{\underset{\begin{array}{l} j=1\\ j\neq i \end{array}}{\sum }}U_{j}B_{ij}\sin\delta_{ij}-D_{i}\omega_{0}\Delta \omega_{i} & \end{array}$$

According to the Lyapunov stability theorem, the energy function *V*(*δ*, *ω*) = *E*_k_ + *E*_p_ is constructed, where *E*_k_ represents the kinetic energy of the system and *E*_p_ represents the potential energy of the system, as specifically shown in Equation (23):

(23) $$\begin{array}{l} E_{k}=\frac{1}{2}\sum_{i=1}^{n}J_{i}\omega_{0}\Delta \omega_{i}^{2}+\frac{\omega_{0}}{2}\sum_{i=1}^{n}J_{xi}{\left(\Delta \omega_{i}-\Delta \omega_{j}\right)}^{2}\mathrm{sign}\frac{d\omega_{i}}{dt}\\ E_{p}=-\sum_{i=1}^{n}\left(P_{Ti}-P_{ei0}\right)(\delta_{i}-\delta_{0})-\sum_{i=1}^{n-1}\sum_{j=1}^{n}U_{i}U_{j}B_{ij}(\cos\delta_{ij}-\cos\delta_{ij0}) \end{array}$$

The constructed Lyapunov energy function *V*(*δ*, *ω*) = *E*_k_ + *E*_p_ includes two distinct energy components with clear physical meanings. Specifically, *E*_k_ represents the kinetic energy stored in the rotating mass of the virtual synchronous generator, reflecting the mechanical-like inertia characteristic of the system, which is analogous to the rotor kinetic energy of a traditional synchronous machine. On the other hand, *E*_p_ corresponds to the potential energy associated with electromagnetic interactions and power exchanges between the VSG units and the electrical network, representing the electromagnetic energy stored due to phase-angle deviations from the equilibrium point. Thus, the decrease in the total energy *V*(*δ*, *ω*) over time ensures that the system oscillations decay, indicating stable system operation under the proposed control strategy.

The derivative of the energy function is expressed as Equation (24). From Equation (24), it can be concluded that the additional inertia control proposed in this paper is stable when the damping coefficient *D_i_* is greater than 0. Moreover, during the steady-state operation of the system, the additional inertia term is Δ*J_i_* = 0. Therefore, the additional inertia control proposed in this paper does not affect the steady-state operation process and only suppresses frequency oscillations during transient processes:

(24) $$\begin{array}{l} \dot{V}(\delta ,\omega )=\frac{dV}{dt}=\frac{dE_{k}}{dt}+\frac{dE_{p}}{dt}\\ =\sum_{i=1}^{n}J_{i}\omega_{0}\Delta \omega_{i}\frac{d\omega_{i}}{dt}+\sum_{i=1}^{n}J_{\mathrm{xi}}\omega_{0}(\Delta \omega_{i}-\Delta \omega_{j})\mathrm{sign}\frac{d\omega_{i}}{dt}\Delta \omega_{i}\frac{d\omega_{i}}{dt}\\ -\sum_{i=1}^{n}\left(P_{Ti}-P_{ei0}\right)\Delta \omega_{i}-U_{i}\overset{j=n}{\underset{\begin{array}{l} j=1\\ j\neq i \end{array}}{\sum }}U_{j}B_{ij}\sin\delta_{ij}\Delta \omega_{i}\\ =\sum_{i=1}^{n}(J_{i}+J_{xi}(\Delta \omega_{i}-\Delta \omega_{j})\mathrm{sign}\frac{d\omega_{i}}{dt})\omega_{0}\Delta \omega_{i}\frac{d\omega_{i}}{dt}\\ -\sum_{i=1}^{n}\left(P_{Ti}-P_{ei0}\right)\Delta \omega_{i}-U_{i}\overset{j=n}{\underset{\begin{array}{l} j=1\\ j\neq i \end{array}}{\sum }}U_{j}B_{ij}\sin\delta_{ij}\Delta \omega_{i}\\ =\sum_{i=1}^{n}\left(P_{Ti}-P_{ei0}\right)\Delta \omega_{i}-U_{i}\overset{j=n}{\underset{\begin{array}{l} j=1\\ j\neq i \end{array}}{\sum }}U_{j}B_{ij}\sin\delta_{ij}\Delta \omega_{i}-\sum_{i=1}^{n}D_{i}\omega_{0}\Delta \omega_{i}^{2}\\ -\sum_{i=1}^{n}\left(P_{Ti}-P_{ei0}\right)\Delta \omega_{i}+U_{i}\overset{j=n}{\underset{\begin{array}{l} j=1\\ j\neq i \end{array}}{\sum }}U_{j}B_{ij}\sin\delta_{ij}\Delta \omega_{i}\\ =-\sum_{i=1}^{n}D_{i}\omega_{0}\Delta \omega_{i}^{2}<0 \end{array}$$

## 5. Simulation Verification

**Figure 5:**
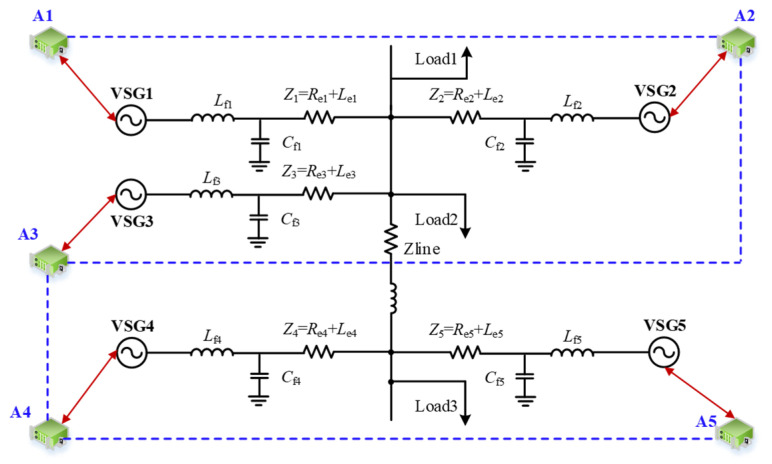
Simulation diagram of multi-VSG parallel system.

To verify the effectiveness of the proposed virtual inertia control strategy, a parallel system of five VSGs in an islanded mode was built in MATLAB/Simulink (Version: R2022a). The topology of the system is shown in Figure 5, and the simulation parameters are listed in Table 1 and Table 2. The solid black lines in the topological diagram represent power circuits, while the blue dashed lines indicate communication links.

### 5.1. Comparison with the Unimproved VSG Control Strategy

**Figure 6:**
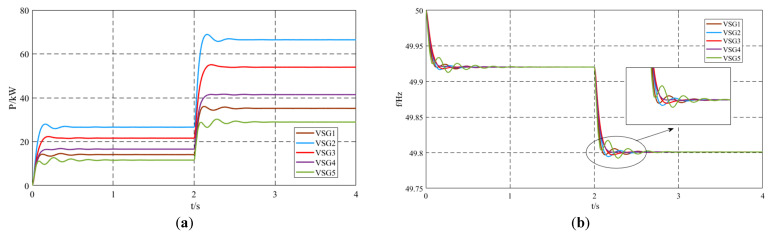
Simulation results of the unimproved control strategy: (**a**) output active power; (**b**) output frequency.

Five VSG units operated in parallel. At *t* = 0 s, Load1 = 100 kW + 50 kvar was connected, and the system operated under this condition for 2 s. At *t* = 2 s, Load2 = 200 kW + 100 kvar was added. The active power and frequency output using the unimproved control strategy are shown in Figure 6. Under the conventional control strategy, significant oscillations in the output active power and frequency were observed. System frequency significantly affected the power quality of the grid. During normal load switching, due to the poor dynamic performance of the system, the frequency oscillations were severe, which can harm the power quality of the grid and affect its stability. In severe cases, it may lead to system frequency collapse.

**Figure 7:**
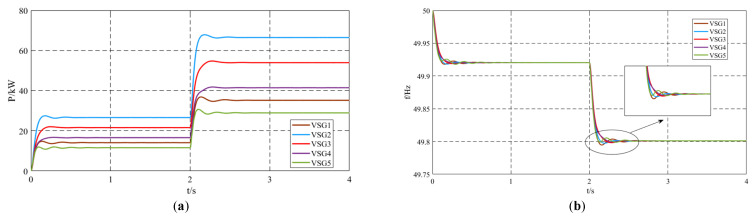
Simulation results of virtual inertia control strategy: (**a**) output active power; (**b**) output frequency.

Ignoring the influence of the damping coefficient on the improved inertia control strategy, the damping coefficient for all units was set to *D_i_* = 10, and the interaction inertia coefficient for all units was set to *J_mi_* = 150. The remaining settings were the same as those of the unimproved control strategy. The simulation results are shown in Figure 6. When the system adopts the virtual inertia control strategy, the oscillations in active power and frequency are significantly suppressed. Comparing the output active power in Figure 6a and Figure 7a, when Load2 is connected to the system, the overshoot of active power oscillation with the unimproved control strategy is 3.6%, while with the virtual inertia control strategy, it is 2%. The oscillation time for active power with the unimproved control strategy is 1.07 s, whereas with the virtual inertia control strategy, it is 0.67 s, indicating an improvement in the system’s dynamic performance.

Comparing the output frequencies in Figure 6b and Figure 7b, when Load2 is connected at *t* = 2 s, the maximum frequency without the control strategy is 49.8174 Hz, while with the control strategy, it is 49.8052 Hz. The minimum frequency without the control strategy is 49.7924 Hz, while with the control strategy, it is 49.7988 Hz. The final steady-state frequency in both cases is 49.801 Hz. The deviation from the maximum frequency with the control strategy is reduced by 74.39% compared to the case without the control strategy, and the deviation from the minimum frequency with the control strategy is reduced by 74.42% compared to the case without the control strategy. By applying the proposed virtual inertia control strategy during the frequency oscillation process, the deviation between the extreme frequency values and the steady-state frequency is significantly reduced. Additionally, the frequency oscillation time is reduced. Without the control strategy, the output frequency process lasts for three oscillation cycles with a duration of 0.8 s, while with the virtual inertia control strategy, the output frequency oscillation is significantly suppressed after only one oscillation cycle, with a duration of 0.39 s. The detailed quantitative comparison of the simulation results between the unimproved control strategy and the proposed virtual inertia control strategy is summarized in Table 3.

**Figure 8:**
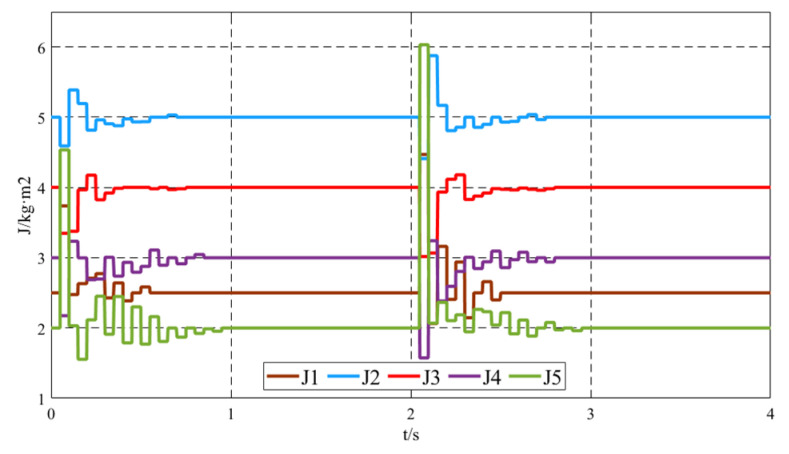
Virtual inertia *J* variation curve.

Figure 8 shows the virtual inertia *J* variation curves of the five VSGs within the 0–4 s time frame. When the system’s load changes, such as during the initial operation phase when Load1 is connected and at *t* = 2 s when Load2 is connected, the improved virtual inertia control strategy comes into effect. Based on the angular frequency deviation between the local VSG and its neighboring VSGs, as well as its own angular frequency rate of change, the virtual inertia of the local unit is adjusted. This ensures that when the frequency deviates from the steady-state value, the virtual inertia is increased to reduce the extent of the deviation, and when the frequency approaches the steady-state value, the virtual inertia is decreased to accelerate the convergence. Under the improved virtual inertia control strategy, the variation of *J* corresponds to the characteristics of frequency oscillations, increasing and decreasing as the oscillation process progresses. However, during steady-state operation, the virtual inertia output of the VSG units is 0, representing the inherent virtual inertia of the units.

### 5.2. Validation of Strategy Effectiveness Under Load Fluctuation Conditions

**Figure 9:**
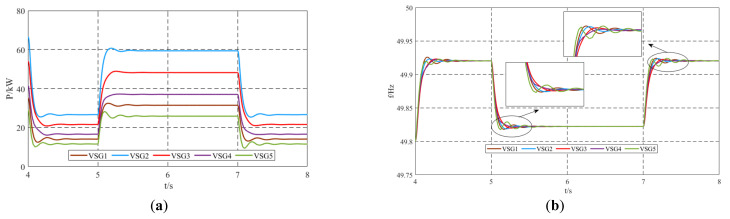
Simulation results under load fluctuation conditions: (**a**) output active power; (**b**) output frequency.

**Figure 10:**
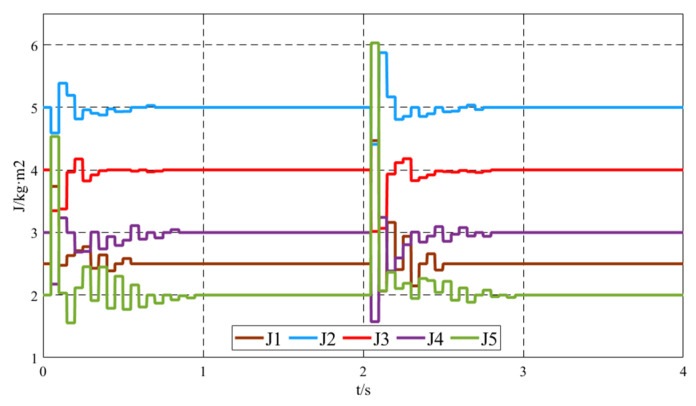
Virtual inertia *J* variation curve under load fluctuation.

Under load fluctuation conditions, specifically when Load2 is disconnected at *t* = 4 s, Load3 = 150 kW + 50 kvar is connected at *t* = 5 s, and Load3 is disconnected at *t* = 7 s, the simulation results of the output active power and frequency using the proposed virtual inertia control strategy are shown in Figure 9. The results demonstrate that the proposed virtual inertia control provides dynamically varying virtual inertia to the system under load fluctuation conditions, effectively suppressing oscillations in the output active power and frequency. The specific changes in virtual inertia are illustrated in Figure 10. Even under load fluctuations, the virtual inertia control strategy dynamically adjusts the virtual inertia of each unit through neighbor communication among the units, highlighting the robustness of the proposed control strategy. After the dynamic process ends, the additional inertia Δ*J_i_* of each VSG approaches zero, and the virtual inertia *J* converges to its preset value, completing the adjustment.

### 5.3. Validation of Strategy Effectiveness Under Communication Delay Conditions

**Figure 11:**
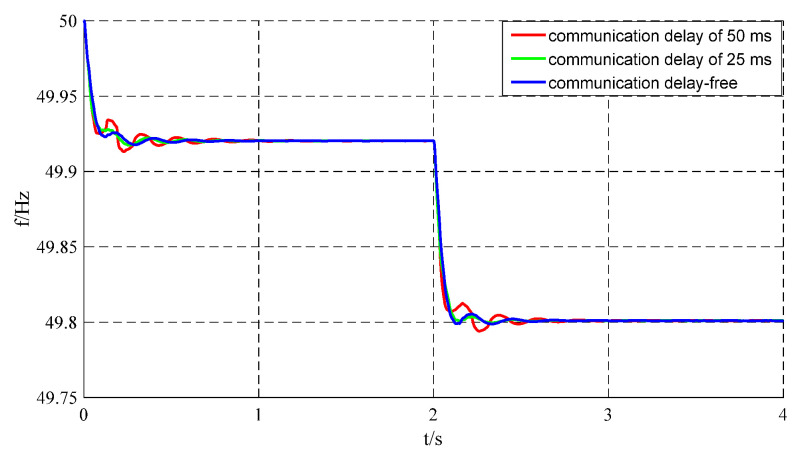
VSG5 frequency waveform under communication delay.

The proposed virtual inertia control strategy in this paper relies on neighbor communication for information exchange, adjusting the local virtual inertia based on neighboring frequency information. Therefore, the effectiveness of this control strategy significantly depends on communication transmission speed. In the MATLAB/Simulink (Version: R2022a) simulation setup, the sampling time was set as *T*_d_ = 50 ms, 25 ms, and 0 ms. Taking VSG Unit 5 as an example and following the simulation scenario format with Load1 connected at 0 s and Load2 at 2 s, the impacts of the three communication delay scenarios on system frequency oscillations under the proposed control strategy are presented in Figure 11.

When the system operates without communication delay, it exhibits minimal frequency oscillations lasting approximately three cycles. With a 25 ms communication delay, the oscillation magnitude increases and persists for four cycles. When the delay increases further to 50 ms, the system shows maximum frequency deviation to 49.9341 Hz with a steady-state frequency of 49.9203 Hz, resulting in a difference of 0.0138 Hz between peak and steady-state frequencies. This represents a 55.6% increase compared to the 0.0054 Hz difference observed in the delay-free scenario. Furthermore, the 50 ms delay case requires about 1 s (approximately six oscillation cycles) to regain stability, whereas the delay-free system stabilizes within just 0.39 s. A detailed summary of the influence of communication delays on frequency oscillation for VSG Unit 5 is presented in Table 4.

## 6. Conclusions

This paper investigates the frequency oscillation issue in multi-VSG parallel systems and proposes a dynamic virtual inertia control strategy based on neighbor communication. First, a detailed state–space small-signal modeling of the multi-VSG parallel system was conducted, demonstrating the system’s small-signal stability. Subsequently, a distributed virtual inertia control strategy based on neighbor communication was proposed, incorporating a frequency observer design. The control strategy utilizes the difference between the local frequency and the average frequency of neighboring units as an adjustment term to regulate the additional inertia component. Furthermore, the stability of the proposed control strategy was rigorously proven through the construction of a Lyapunov energy function based on Lyapunov’s theorem. Finally, simulation results validate the effectiveness of the proposed control method. Compared with unmodified control strategies, the proposed virtual inertia control approach can effectively suppress oscillations in both active power output and frequency while reducing the duration of system oscillations. Notably, this control strategy achieves its objectives with minimal communication requirements.

## Figures and Tables

**Table 1:** Main circuit simulation parameters of multi-machine parallel system.

Parameter	VSG1	VSG2	VSG3	VSG4	VSG5
*L*_f_/mH	250 × 10^−3^	250 × 10^−3^	250 × 10^−3^	250 × 10^−3^	250 × 10^−3^
*C*_f_/F	100 × 10^−6^	100 × 10^−6^	100 × 10^−6^	100 × 10^−6^	100 × 10^−6^
*R*_e_/Ω	0.15	0.15	0.15	0.09	0.05
*L*_e_/mH	0.54	0.32	0.44	0.44	0.38

**Table 2:** Control system simulation parameters of multi-machine parallel system.

Parameter	VSG1	VSG2	VSG3	VSG4	VSG5
*J*/(kg·m^2^)	2.5	5	4	3	2
*J*_m*i*_/(kg·m^2^·s/rad)	150	150	150	150	150
*D*/(kW·s^2^/rad^2^)	10	10	10	10	10
*K*_ω_	2.5 × 10^4^	5 × 10^4^	4 × 10^4^	3 × 10^4^	2 × 10^4^
*K*_Q_	10 × 10^−4^	10 × 10^−4^	10 × 10^−4^	10 × 10^−4^	10 × 10^−4^

**Table 3:** Simulation performance comparison between the proposed strategy and the unimproved strategy (Load1 connected at *t* = 0 s, Load2 connected at *t* = 2 s).

Performance Indices	Unimproved Control Strategy	Proposed Virtual Inertia Control Strategy
Active power overshoot	3.6%	2%
Active power oscillation duration	1.07 s	0.67 s
Maximum frequency (Hz)	49.8174	49.8052
Minimum frequency (Hz)	49.7924	49.7988
Steady-state frequency (Hz)	49.801	49.801
Reduction of max frequency deviation from steady-state	—	74.39%
Reduction of min frequency deviation from steady-state	—	74.42%
Frequency oscillation duration	0.8 s	0.39 s

**Table 4:** Influence of communication delay on frequency oscillation of VSG Unit 5 (Load1 connected at t = 0 s, Load2 connected at t = 2 s).

Performance Indices	No Delay (0 ms)	25 ms Delay	50 ms Delay
Steady-state frequency (Hz)	49.9203	49.9203	49.9203
Maximum frequency (Hz)	49.9257	—	49.9341
Max frequency deviation from steady-state (Hz)	0.0054	—	0.0138
Increase in max frequency deviation (relative to no delay)	—	Slight increase	55.6%
Number of oscillation cycles	3 cycles	4 cycles	6 cycles
Time required for system stabilization	0.39 s	Slightly > 0.39 s	1.0 s

## Data Availability

The data used were shared in our paper.
